# Meaningless Use: Assessing Compliance With a Clinically Meaningless Emergency Department Documentation Requirement

**DOI:** 10.7759/cureus.37244

**Published:** 2023-04-07

**Authors:** Stephen Guilherme, Lisa O Iyeke, Yi-Ru Chen, Aliya Catanzarita, Melva Morales Sierra, Renee Clouden, Daisy Puca, Mark Richman

**Affiliations:** 1 Emergency Medicine, Long Island Jewish Medical Center, New Hyde Park, USA

**Keywords:** regulations, documentation, emergency medicine, professional burnout, meaningful use

## Abstract

Introduction

A New York State initiative requests that Emergency Department (ED) providers document in the electronic health record (EHR) each admitted patient’s employment status and, if applicable, their mode of commute. This initiative diverts them from their primary duties and increases the likelihood they will either disregard the request or input incorrect information to complete the data fields as fast as possible.

This study intends to understand how well providers adhere to this regulation, which, while important for society as a whole, has little clinical relevance, especially in the ED, where the focus is to identify and treat emergent conditions. We hypothesized that clinician-collected employment data would contain many more "N/A" responses than registration-collected employment data (the "gold standard").

Methods

We took a randomly selected convenience sample of 100 patients admitted from the ED and compared each patient’s provider-entered response to the employment data field to the registration-recorded response. The EHR operates such that the "Employment" field must be completed in order to complete the admission electronically. Data fields collected were: last name, first name, date of birth, medical record number, date and time of arrival, date and time of admission, attending physician, resident physician (if there was one), mid-level provider (if there was one), provider-entered employment status, registration-entered employment status, admitting service (eg, Medicine, Surgery, OB/Gyn), and disposition level (eg, ICU). We assessed the percent of employment data that was concordant between the provider's entry and the registration clerk's entry. We also assessed for the potential confounding variable of how busy the ED was at time of admission, as providers may not take ask about employment or enter such data during particularly busy times. Finally, we interviewed providers to elicit reasons they did not enter accurate data. Statistical significance was set a priori at p <0.05.

Results

One hundred six patients were screened; six were excluded because one of the authors (MR) was their attending physician. For 92 of the remaining 100 patients, providers recorded employment as “N/A," and for eight patients they recorded “retired." For seven of these eight patients, provider entry matched registration entry (87.5% concordance). To adjust for whether how busy the ED was may have impacted the accuracy of data entry, admissions were categorized according to what time of day the patient was admitted. There was no statistically significant correlation between how busy the ED was and accuracy of data entry. The majority of providers stated they responded "NA" because the employment information was unrelated to the ED visit.

Conclusion

In New York, for each patient admitted from the ED, the ED provider is requested to enter the patient's job information and, if they commute to work, the method they use. However, this takes providers' attention away from what they should be doing most: diagnosing and treating patients. This study highlights the unintended consequence of requesting data fields that are not clinically relevant and, from the patient and provider perspective, are not good investments of time and energy and distract from the clinical visit. Persons interpreting such clinically irrelevant data should do so with caution, as the results are unlikely to reflect the truth of what the questions intend to determine.

## Introduction

The burnout epidemic among healthcare providers is fueled by: 1) increasing amounts of time focusing on the electronic health record (EHR), such as typing notes, performing computer physician order entry (CPOE)), 2) stagnant pay relative to cost of living, and 3) increasing administrative paperwork, such as forms to be filled out on behalf of patients (disability, work note, pre-employment) and regulatory requirements (screening for depression, domestic violence, smoking, human trafficking, etc.) [1-4]. These regulatory requirements are embedded as EHR data fields. Many required data fields are irrelevant to the clinician’s task at hand: providing good medical care. Such requirements fuel burnout and resentment, and, as such, run the risk of either being ignored by clinicians, or containing inaccurate data, as the clinician simply bypasses or quickly enters inaccurate data. 

In 2019, New York State began an effort to collect the employment status and commute method for admitted patients. Emergency Department (ED) providers were encouraged to record in the EHR whether the patient is employed, and, if so, their means of commuting to work. This initiative was voluntary for hospital systems to comply with; some adopted the initiative and modified their EHRs to include ED data entry fields for admitted patients to include employment and mode of transportation to such employment. 

Northwell Health, the largest private employer in New York State, adopted the initiative and adapted its Allscripts® EHR ED module to capture employment and commute data. Providers are required to respond to the "Employment" “screening” prompt by selecting at least one radio button response option: Unemployed, Disabled, Retired, Homemaker, Employed, Self-employed, Student, N/A. If “Employed” or “Self-employed” is selected, a narrative data entry field appears asking “Occupation,” followed by a second radio button option list with options: Public Transportation, Personal Automobile, Car Service/Taxi, Walking, Biking, N/A (Working From Home), and Other. Providers cannot proceed with admitting the patient without completing this process.

While it is common for providers to inquire about patients’ employment (as it may be relevant to their chief complaint), it is uncommon to inquire about how they commute to work. Requesting such data from busy clinicians distracts from their main job and is prone to either be ignored or result in clinicians entering inaccurate data so as to quickly get through those data fields. Consequently, compliance with such a requirement might be expected to be quite low.

The primary aim of this quality improvement study was to determine clinicians’ compliance with this regulatory requirement which, while important for the broader society, is clinically meaningless, particularly in a setting aimed to assess and address emergent conditions (ie, the ED). We hypothesized that, compared to registration-collected employment data, clinician-collected employment data would have many more answers as “N/A." That is, for the same patient, the clinician might enter "N/A," while the registration data field recorded the patient was, indeed, employed. The secondary aim was to investigate reasons providers did not enter accurate data.

## Materials and methods

Long Island Jewish (LIJ) Medical Center is a 583-bed tertiary-care academic hospital, serving a racially and socio-economically diverse population. We took a randomly selected convenience sample of 100 patients admitted from the ED, none of whom was admitted by any of the authors, and compared each patient’s physician-entered response to the employment data field to the registration-recorded response to the employment field. The EHR operates such that the "Employment" field cannot be skipped (ie, there must be data entered into that field) in order to progress with completing the admission electronically. The registration data was considered to be the “gold standard.” Every patient (or their family member, if the patient is unable to speak), is approached by registration and asked: 1) whether they are employed, 2) their specific job, and 3) who their employer is. This data is entered in a separate application, which is imported into the main EHR, but stored in a place separate from the clinician-facing employment question. Because this process is extremely standardized and is tied to the ability to achieve reimbursement for the visit, it is performed consistently and, therefore, is the “gold standard” for collection of employment data.

Patients were included if they were admitted from LIJ’s ED to non-psychiatric services (eg, Internal Medicine, General Surgery) between October 1, 2022 and December 31, 2022. Patients were excluded if the patient's ED attending physician was an author of this manuscript (MR); none of the other authors were in a position to admit patients.

Data fields collected were: last name, first name, date of birth, medical record number, date of arrival, time of arrival, date of admission, time of admission, attending physician, resident provider (if there was one), mid-level provider (if there was one), provider-entered employment status, registration-entered employment status, admitting service (eg, Medicine, Surgery, OB/Gyn), disposition level (eg, medical/surgical floor, ICU).

During busy ED times (typically, 12 PM-midnight) [5], providers are less likely to document thoroughly [6]; this is likely even more so as pertains to non-clinically relevant documentation, such as employment or commute data. To adjust for whether this may have had an impact on the accuracy of data entry, the admissions were categorized according to what time the patient was admitted; those admitted between 12 PM and 11:59 PM were considered to have been admitted during a typically “busy” time, while those admitted between 12 AM and 11:50 AM were considered to have been admitted during a typically "not busy” time. An analysis was performed to determine whether there was an association between how busy the ED was and likelihood to enter accurate information. As our EHR cannot retrospectively capture ED status/busyness metrics (eg, treatment, waiting, and boarding volume; wait and boarding times) at specific times in the past, we were unable to utilize these metrics to determine whether there was a correlation between busyness and accuracy of the data that was entered.

We sampled providers who did not enter accurate information to determine their reason for not doing so. Fifteen attending physicians, 12 resident physicians, and 18 physician assistants were asked the standard questions: 1. Do you routinely enter data in the “Employment” data field? If they answer in the negative, they were asked (in a non-judgmental manner) “Why do you not enter such information?”

Statistical significance was set a priori at p <0.05. 

This study was deemed exempt by the Institutional Review Board (IRB #: 23-0153).

## Results

For the 100 admitted patients included in the study, providers recorded the employment of 92% as “N/A,” while the remaining 8% were all entered as “retired.” Of these eight patients, seven (87.5%) were concordant with the registration data. Overall, only 7% of provider-entered records matched those of registration (p <0.0001). None of the 37 attendings or three physician assistants associated with the patient admissions entered any employment data, while 5.9% of the associated residents (two of 34) did. Among the 100 patients, 81 were noted by registration to be unemployed, retired, or unknown. Meanwhile, 16 were identified as employed, one self-employed, one student and one home-maker. Of the entries which matched with registration, seven were recorded by the same physician, while the one remaining entry, which did not match, was recorded by another physician. 

To adjust for whether how busy the ED was impacted provider-entered data accuracy, admissions were categorized according to what time the patient was admitted; those admitted between 12 PM and 11:59 PM were considered to have been admitted during a typically “busy” time, while those admitted between 12 AM and 11:50 AM were considered to have been admitted during a typically “non-busy” time. There was no statistically significant correlation between time of admission and accuracy of data entry (p = 0.1201).

Providers’ stated reasons for not entering data in the field were nearly unanimous: the information requested in the field is (variably described as) “irrelevant,” “not applicable,” “not related to their ED visit,” “not important to their medical care,” etc. One provider was concerned the data would be used in a manner detrimental to patients (discrimination on the basis of employment or means of commute, which might be a surrogate for ability to pay or to access care).

## Discussion

In 2011, the United States government, via the Office of the National Coordinator for Health Information Technology (ONC), released its “meaningful use” criteria for determining whether an EHR was functional and added value (worth reimbursing through Medicare and Medicaid payments from the EHR incentive program) [7]. Individual physicians and other eligible healthcare professionals could each receive up to $44,000 through the Medicare Meaningful Use program or up to $63,750 through the Medicaid Meaningful Use program, depending upon when they begin attesting to the program's requirements [8]. Examples of meaningful use include decision support rules based on laboratory results and ability for patients to access online clinical information. However, since then, many not-clinically meaningful data fields have been added to EHRs. This has been facilitated by: 1. the increasing number of clinical and non-clinical organizations who desire data, 2. the ease of adding data fields, and 3. the ease of extracting or exporting data from EHRs. The unintended consequence of this proliferation of data fields is that many are not clinically relevant and, from the patient and provider perspective, are not good investments of time and energy and distract from the clinical visit. Such clinically meaningless administrative/regulatory tasks are linked causally with burnout [9]. Burnout, in turn, is associated with lower quality of care, medical errors, reduced productivity and access, and poor physician mental and physical health [10]. Completing each individual data field may not take much time, but doing this for many fields and many patients creates substantial cumulative burden. Such expenditures of time and mental energy also increase providers’ sense of powerlessness in controlling their work environment or equipment (eg, EHR).

Not surprisingly, then, this investigation found that in only 8% of admissions was clinically meaningless employment data entered at all by the provider, even after adjusting for how busy the ED was at the time of admission. In only 7% of admissions were the employment data concordant between the provider and registration. This suggests such data is irrelevant to the ED provider, whose focus is stabilizing and diagnosing the patient and making a timely discharge or admission disposition. In those instances data was entered, it was accurate (correlated well in nearly 90% of instances compared with the “gold standard” of registration-acquired information). Accurate data entry appears to be provider-dependent (ie, based on a provider’s commitment to thorough and accurate charting and fulfillment of mandated data fields), rather than situation-dependent. If the impetus to enter data was situation-dependent, one might expect more of the attendings, residents, and PAs to have, at least once, entered data.

Actions unnecessary to clinical care distract from providers’ main concern: high-quality medicine. Such distractions are common and associated with delays in care and medical errors [10]. Evidence of the effect of similar clinically non-actionable behavior can be found analogously in the study of alarm fatigue. Device alarms (eg, blood pressure, heart rate) are intended to notify healthcare staff of clinically meaningful changes in a patient’s condition. However, many such alarms are non-actionable (ie, do not require immediate attention). Actionable alarms account for only 5-13% of alarms in current monitoring systems [11]. Persistent distraction from clinically non-actionable alarms promotes alarm fatigue, which consists of two components: alarm desensitization from sensory overload and blunting of responsiveness, and alarm apathy in which oversensitive and under-specific alarms decrease trust in their validity [12]. There is a dose-response relative to alarm exposure and fatigue. As the number of non-actionable alarms increases, there is an incremental increase in clinician response delay [13,14].

While providers are overburdened by third-party administrative documentation (eg, pharmaceutical or MRI prior authorization forms), such documentation contains clinical material and can be understood as part of a process of advocating for something clinically meaningful (medications or imaging). In contrast, employment and transportation/commute data for external agencies contains no clinical material and serves no overt clinical purpose.

Non-clinical use of EHRs is a consistent source of frustration and contributor to burnout. According to the National Physician Poll by Harris Polls titled “How Doctors Feel About Electronic Health Records,” only 8% of primary care physicians say the primary value of their EHR is clinically related; 40% of PCPs believe there are more challenges with EHRs than benefits; and ~50% think using an EHR detracts from their clinical effectiveness. The large amount of time (62%) spent with the EHR rather than interacting with the patient has led ~70% to agree EHRs greatly contribute to burnout [15].

The situation is well-recognized, to the point the Office of the National Coordinator for Health Information Technology published in 2020 the Strategy on Reducing Regulatory and Administrative Burden Relating to the Use of Health IT and EHRs with the goals of: 1) reducing the effort/time to record EHR information 2) reducing the effort/time to meet regulatory reporting requirements, and 3) improving the functionality and intuitiveness of EHRs [16]. Recommendation #1 is “Reduce regulatory burden around documentation requirements for patient visits,” with potential strategies such as “stakeholders should work to ensure that relevant information already captured by other care team members is easily accessible to the clinician in the electronic record.” In the case of New York State’s employment documentation initiative, this data could be abstracted to New York State from the more reliable registration database; or, at minimum, exported from that database into the providers’ EHR, from whence it could be exported to New York State. 

Adding social determinants of health (SDoH) into the EHR has been demonstrated to facilitate referrals to community resources [17], which, in turn, are associated (in some studies) with improved outcomes in the domains of experience of care (eg, change in social needs, patient satisfaction) and population health (eg, diet quality, blood cholesterol levels) [18]. Exporting data from non-healthcare entities to an EHR is possible, and several such programs are in place. Community-based organizations in Texas [19] and Michigan [20] are integrating SDoH information into the statewide health information exchange (HIE), from whence such data can be exported into EHRs.

Secondary use of the EHR occurs when the EHR, or parts of it, are used for purposes other than those that for which it was created (clinical, medico-legal, or financial). Adding non-clinical data fields (such as "Employment") for other purposes is an example of secondary use. Secondary use of EHR data is prone to issues of quality. In their study of the quality of secondary data abstracted for a cohort of patients with pancreatic cancer, Botsis et al. identified three domains of data problems: incompleteness, inconsistency, and inaccuracy [21]. Such issues are common and plague data abstraction for both research and clinical purposes. An evaluation of smoking history data, which determines eligibility for low-dose CT scanning for lung cancer, found 80% of evaluated records had inaccuracies [22]; patients may either be inappropriately screened, or not screened when they should be. Providers are prone to inaccurate data entry even in high-risk situations; one intensive care unit study of reasons providers override medication warnings/alerts found they chose “inaccurate warning/alert” ~25% of the time when, in fact, the alerts were accurate. Such misrepresentations could lead future providers to order inappropriate medications [23].

Consequently, another important implication of this study's findings is that those responsible for creating clinically meaningless data fields and collecting, analyzing, and interpreting data from such fields should view the results they receive with caution. The results may not represent truth/reality and may lead to ineffective policy, operations, procedure, budget, and staffing decisions. For example, policymakers using provider-entered data might conclude 0% of LIJ ED’s patients are employed if they assume an “N/A” entry indicates “unemployed” and combine that with the remaining 8% listed as “retired.” In fact, 35% were employed (19 as employees and 16 as “self-employed”) and 54% were retired, which might lead to a different set of policy or financial allocation decisions.

Limitations

This was a study in a single department at a single site. ED volume or the number of critical patients might have affected how willing providers would have been to fill in extraneous data. However, we attempted to adjust for this using a surrogate marker for how busy the ED was: whether the time of admission was during a typically busy time (12 PM - 11:59 PM) or not (12 AM - 11:59 AM). These categorizations are crude, as the ED (and, in particular, a specific provider) might be busy or have a heavy caseload during typically non-busy times, and vice versa. We chose this method of estimating how busy the ED was because other measures would have been challenging to obtain; it is difficult to determine how many patients a provider had at a particular time (eg, at 10:27 AM, Provider A was caring for X # of patients). However, how busy the ED is seems unrelated to the commitment to entering accurate data, as only two of 74 providers (2.9%) entered data at all, and there was no statistically significant relationship between time of admission and accuracy of data entry.

## Conclusions

New York State requests that, for each admitted patient, the ED provider must record in the EHR their employment, and, if they work, how they commute to work. Such information can be societally valuable, informing hospital policies and procedures, and local municipal, regional, and federal work and transportation policies and resource distribution. For example, if a substantial portion of a hospital catchment area's patients are unemployed, then the hospital's "social determinants of health" burden will likely be higher, and the hospital may require additional funding for social workers, linkages to community-based non-profit organizations, etc. Likewise, if a large proportion of a hospital's employed patients commute by private automobile, then the region may require more public transit. However, such employment and commute documentation distracts providers from their main concern of diagnosing and treating patients. We discovered the vast majority of clinicians chose “N/A” in response to the "employment" question because providers believe such information is “irrelevant,” “not applicable,” “not related to their ED visit,” “not important to their medical care,” etc. This study highlights the unintended consequence of requesting data fields that are not clinically relevant and, from the patient and provider perspective, are not good investments of time and energy and distract from the clinical visit. If health system leadership demonstrated to providers evidence that such data collection confers tangible, clinically meaningful benefits to patients and providers (eg, funding for an ED social worker), providers might be persuaded to record more accurate data. Persons interpreting such clinically irrelevant data should do so with caution, as the results are unlikely to reflect the truth of what the questions intend to determine. 
